# Advancing Dentistry through Bioprinting: Personalization of Oral Tissues

**DOI:** 10.3390/jfb14100530

**Published:** 2023-10-20

**Authors:** Dobromira Shopova, Anna Mihaylova, Antoniya Yaneva, Desislava Bakova

**Affiliations:** 1 Department of Prosthetic Dentistry, Faculty of Dental Medicine, Medical University of Plovdiv, 4000 Plovdiv, Bulgaria; 2 Department of Healthcare Management, Faculty of Public Health, Medical University of Plovdiv, 4000 Plovdiv, Bulgariadesislavabakova@gmail.com (D.B.); 3 Department of Medical Informatics, Biostatistics and eLearning, Faculty of Public Health, Medical University of Plovdiv, 4000 Plovdiv, Bulgaria; yaneva.antonya@gmail.com

**Keywords:** bioprinting, review, personalized dentistry, regenerative dentistry, stem cells, pulp-dentin regeneration, bone regeneration, periodontium regeneration, gingival regeneration

## Abstract

Despite significant advancements in dental tissue restoration and the use of prostheses for addressing tooth loss, the prevailing clinical approaches remain somewhat inadequate for replicating native dental tissue characteristics. The emergence of three-dimensional (3D) bioprinting offers a promising innovation within the fields of regenerative medicine and tissue engineering. This technology offers notable precision and efficiency, thereby introducing a fresh avenue for tissue regeneration. Unlike the traditional framework encompassing scaffolds, cells, and signaling factors, 3D bioprinting constitutes a contemporary addition to the arsenal of tissue engineering tools. The ongoing shift from conventional dentistry to a more personalized paradigm, principally under the guidance of bioprinting, is poised to exert a significant influence in the foreseeable future. This systematic review undertakes the task of aggregating and analyzing insights related to the application of bioprinting in the context of regenerative dentistry. Adhering to PRISMA guidelines, an exhaustive literature survey spanning the years 2019 to 2023 was performed across prominent databases including PubMed, Scopus, Google Scholar, and ScienceDirect. The landscape of regenerative dentistry has ushered in novel prospects for dentoalveolar treatments and personalized interventions. This review expounds on contemporary accomplishments and avenues for the regeneration of pulp—dentin, bone, periodontal tissues, and gingival tissues. The progressive strides achieved in the realm of bioprinting hold the potential to not only enhance the quality of life but also to catalyze transformative shifts within the domains of medical and dental practices.

## 1. Introduction

The field of tissue engineering has experienced recent applications of bioprinting, a technique that enables the precise placement of cells encapsulated within supportive bioinks, thereby constructing intricate scaffolds utilized for the reparation of targeted tissues [1]. This entails essential considerations encompassing the influence of scaffold materials and geometry on the regeneration of dental tissues, the imperative incorporation of dental cells in these applications, and the pivotal role of signaling molecules in the fabrication of clinically pertinent bioengineered dental implant tissues [2].

**Figure 1:**
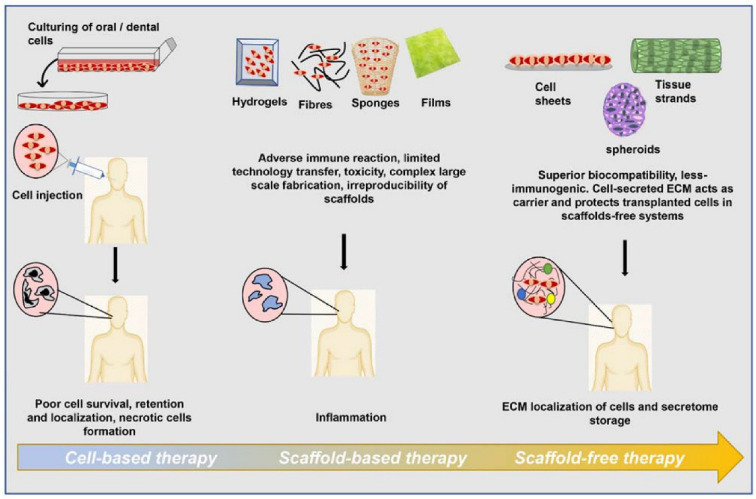
Time-trend transition of cell-based, scaffold-based and scaffoldless therapies used for tissue engineering and regenerative medicine [5].

A novel frontier in tissue engineering is presented by four-dimensional (4D) bioprinting, which introduces the dimension of time as the fourth dimension in conjunction with three-dimensional (3D) bioprinting [3]. The overarching process for 3D bioprinting of dental tissues encompasses several steps: initial 3D modeling based on digital scans of the defect or target area, isolation and differentiation of stem cells into specialized dental tissue cells, formulation and loading of bioink into the bioprinter, actual bioprinting of the desired structure, and subsequent architectural adjustments or chemical enhancements before implantation [4]. The realm of dental biomaterials and regenerative engineering has witnessed substantial advancements, leading to various therapeutic approaches categorized into scaffold-based and scaffold-free methodologies, as depicted in Figure 1. Scaffold-based strategies involve the use of cells, signaling agents, biodegradable materials, and durable polymer frameworks designed to meet specific clinical needs and promote improved cell retention and viability. Nevertheless, challenges such as scientific ambiguity, scalability, cost considerations, regulatory approvals, and technology transfer from laboratory settings to clinical implementation necessitate alternative approaches aligned with regulatory frameworks to attain reproducible clinical outcomes [5].

**Figure 2:**
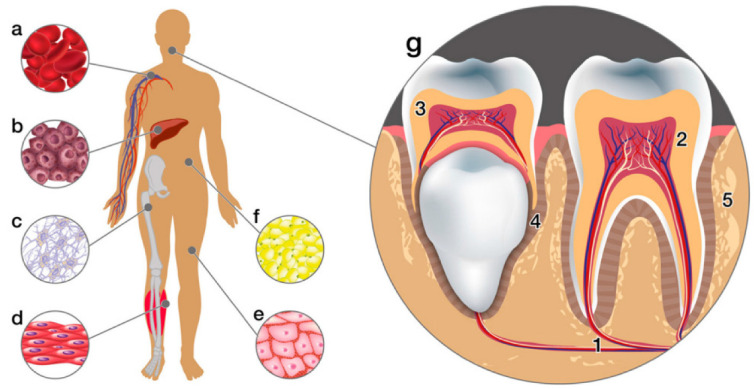
The human organism has various sources of stem cells. The diagram illustrates several tissue sources of adult stem cells, including: (**a**) peripheral blood, (**b**) liver, (**c**) bone marrow, (**d**) muscles, (**e**) skin, (**f**) adipose tissue, and (**g**) dental tissues: (1. apical dental papilla, 2. dental pulp, 3. pulp from the exfoliated deciduous tooth, 4. periodontal ligament, 5. alveolar bone) [11].

The use of automated bioprinting methodologies affords a degree of accuracy in the organization of cells and biological moieties, facilitating the customization of geometrically tailored patient-specific constructs in the domain of biological scaffolds. However, the selection of an appropriate substrate material to encapsulate cells in the formulation of bioinks, designed for bioprinting dentoalveolar tissues, remains a formidable challenge [6]. The integration of bioinks facilitates the investigation of geometric influences and spatial organizations on cellular behavior and functionality within in vitro settings, thus paving the path for subsequent translation into in vivo models applicable to regenerative dentistry [7]. These living cellular constituents require procurement from living organisms. The preeminent cellular source, often employed, is mesenchymal stem cells (MSCs). These cells manifest three pivotal attributes that confer an optimal profile for tissue regeneration endeavors. Primarily, their immunoregulatory capacity serves to mitigate aberrant immune responses. In addition, their paracrine or autocrine functions engender the secretion of growth factors, further augmenting the regenerative processes. Lastly, their propensity to differentiate into target cell lineages underscores their regenerative potential [8,9]. Dental MSCs, in particular, hold profound significance as a reservoir of stem cells within regenerative medicine, offering substantial therapeutic prospects not only within oral pathologies but also extending to diverse extraoral maladies [10]. An ancillary merit of MSCs is their broad availability from an array of adult tissue sources, such as bone marrow, adipose tissue, cutaneous regions, and orofacial tissues, Figure 2 [11].

### 1.1. Scaffold-Free Bioprinting and Stem Cell Diversity in Dentistry

Scaffold-free bioprinting methods have found application as an innovative approach for generating tissue-engineered constructs. These methodologies involve the bioprinting of cell aggregates, typically in the form of spheroid structures. These spheroids can be meticulously positioned to create intricate architectures, such as tubular or ring-like formations [12,13]. Although these constructs are predominantly scaffold-free, the cells are often ensconced within biocompatible and biodegradable hydrogel materials [14,15].

Within the domain of dentistry, two prominent categories of stem cells warrant discussion: embryonic stem cells and somatic (adult) stem cells. Although the use of human embryonic stem cells is fraught with ethical controversies, preliminary investigations are underway for practical therapeutic applications [16]. Dental tissues, with ample potential as cellular reservoirs, encompass the apical dental papilla, dental pulp, exfoliated deciduous tooth pulp, periodontal ligament, and alveolar bone [17]. Indeed, it is worth noting that mesenchymal stem cells with immunosuppressive properties can be easily obtained from dental pulp [18]. DPSCs boast ease of procurement, high plasticity, and multipotential capabilities, and hold promise for low-risk autologous therapeutic interventions aimed at rectifying bodily defects [19].

Human dental pulp stem/progenitor cells (hDPSCs) present an attractive proposition for regenerative therapies because of their amenability to substantial expansion and the generation of colony-forming unit–fibroblasts (CFU-Fs) [20]. Notably, transplantation outcomes employing SBP-DPSCs exhibit great potential for therapeutic utilization, displaying successful transformation into mature bone tissue with complete vascularization [21].

Accessible sources of dental pulp tissue from human third molars, exfoliated deciduous teeth, or supernumerary teeth have emerged as promising repositories for harvesting MSCs. These discarded teeth present an accessible and abundant supply for MSC-based therapies and tissue engineering endeavors [22,23]. Certainly, it is worth mentioning that there has been extensive characterization and comparison between human exfoliated deciduous teeth (SHED) and dental pulp stem cells (DPSCs) [24]. Additionally, postnatal human DPSCs have shown the ability to replicate a dentin/pulp-like complex [25].

### 1.2. Dental Stem Cell Diversity and Regenerative Dentistry Prospects

Karbanova’s research indicates that dental pulp stem cells (DPSCs) exhibit self-renewal capabilities and express markers associated with bone, cartilage, vascular, and neural tissues, suggesting their multipotential attributes [26]. Similarly, Mori’s findings underscore the diverse stemness of dental follicle stem cells (DFSCs), which exhibit the ability to differentiate into osteoblast-like cells capable of producing mineralized matrix nodules while expressing osteoblastic markers [27]. The dental follicle, an encompassing connective tissue, could serve as a repository for mesenchymal stem cells, particularly from impacted teeth that are typically discarded as medical waste [28].

Moreover, the dental follicle has the capacity to differentiate into the periodontal ligament and has the potential to serve as a precursor for various other periodontal cell types, such as osteoblasts and cementoblasts [29,30]. Sonoyama’s work highlights the unique attributes of the apical papilla, which possesses fewer cellular and vascular elements compared to dental pulp but displays robust proliferation in organ cultures [31]. In a comparative analysis, dental stem cells (DSCs) from various sources were characterized, including dental pulp, periodontal ligament, apical papilla stem cells (APSCs), dental follicle stem cells (DFSCs), and bone marrow-derived stem cells (BMSCs). Notably, APSCs and DFSCs demonstrated higher proliferative potentials than BMSCs [32].

In the realm of bone tissue formation, research has emphasized the role of the host organism alongside donor MSCs in the process. This understanding has implications for novel therapeutic strategies that pharmacologically manipulate endogenous MSCs for bone regeneration [33].

**Figure 3:**
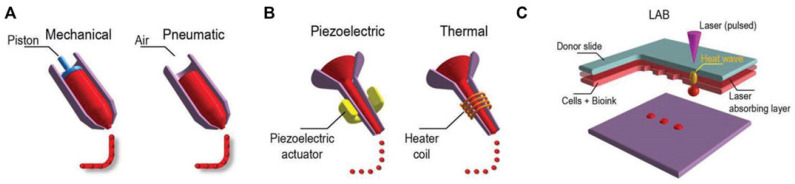
Major techniques in bioprinting: (**A**) Extrusion-based printers utilize mechanical or pneumatic dispensing systems to push out bioink in the form of extruded material; (**B**) Inkjet printers, on the other hand, employ either a pulsed heater that heats the print head, creating air bubbles to force bioink droplets out of the nozzle, or a piezoelectric actuator that generates localized pressure using ultrasonic waves to eject bioink droplets; (**C**) Laser-assisted bioprinters (LABs) utilize a laser beam directed at an absorbing substrate to generate heat waves, which are then used to dispense bioink onto a substrate [1].

There are three major techniques used in bioprinting, Figure 3: 1—extrusion-based printers: these printers utilize mechanical or pneumatic systems to push or extrude a bioink material out of a nozzle onto a substrate; 2—inkjet printers: inkjet bioprinters use either a pulsed heater or a piezoelectric actuator to create localized pressure, which forces tiny droplets of bioink out of the nozzle onto a substrate; and 3—laser-assisted bioprinters (LABs): LABs use a laser beam focused on an absorbing substrate to create heat waves, facilitating the controlled dispensing of bioink onto the substrate. These techniques play a crucial role in the realm of bioprinting, enabling precise deposition of biological materials for a wide range of applications, including tissue engineering and regenerative medicine [1].

The dental apparatus encompasses both hard and soft dental tissues, including alveolar bone, dental pulp, teeth, periodontal ligament, and gums, as well as their associated blood vessels and nerves. This diversity paves the way for regenerative dentistry tailored to each specific structure. This article’s objective is to present contemporary accomplishments and opportunities in the realms of pulp-dentin, bone, periodontium, and gingival regeneration, all integral components of the broader landscape of regenerative dentistry.

## 2. Materials and Methods

This article encompasses a systematic review aimed at exploring the potential applications of bioprinting within the domain of regenerative dentistry. This study follows the guidelines outlined in the Preferred Reporting Items for Systematic Reviews and Meta-Analyses (PRISMA). The ensuing section outlines the materials and methodologies adopted for this review, in alignment with the stipulated PRISMA guidelines [34].

### 2.1. Literature Search

A detailed investigation of the scientific literature was conducted in PubMed, Scopus, Google Scholar, and ScienceDirect databases to identify English-language articles published between 2019 and 2023. The search terms and queries ((3D bioprinting) OR (bioprinting)) AND ((dentistry) OR (regenerative dentistry)) were used to retrieve articles related to bioprinting applications in the field of regenerative dentistry. Additionally, the more precise search terms and queries ((3D bioprinting) OR (bioprinting)) AND ((stem cells) OR (tissue engineering) OR (apical dental papilla) OR (periodontal ligament) OR (dentin) OR (alveolar bone)) were used to identify articles focusing on bioprinting applications within specific dental subdomains.

### 2.2. Eligibility Criteria

The inclusion criteria for articles encompassed the following: (1) publications dated between 2019 and 2023; (2) original articles including reviews, systematic reviews, meta-analyses, randomized controlled trials (RCTs), and observational studies; (3) full-text English-language publications; and (4) studies involving human-derived or animal cells relevant to dental tissue engineering.

The exclusion criteria, on the other hand, were as follows: (1) abstracts, (2) short communications, (3) patents and policy-related manuscripts, (4) case reports, (5) studies that lacked fundamental information about bioprinting, and (6) paid for readers’ articles.

### 2.3. Data Items

In the context of our systematic review, our primary outcome variables were as follows:

- Types of Bioprinting Applications in regenerative dentistry.
- Bioprinting Materials commonly used in bioprinting for regenerative dentistry, encompassing biocompatible polymers, bioinks, and scaffold materials.
- Bioprinting Techniques utilized in regenerative dentistry.
- Dental Tissues and Structures: Our review focuses on the specific dental tissues and structures that have been the main points of bioprinting applications, encompassing dentin, pulp, bone, periodontal tissues, and gingival soft tissues.

### 2.4. Data Analysis

To conduct a rigorous data analysis, a structured data extraction form was created using Microsoft Office Excel 2016. This form was instrumental in maintaining consistency throughout the extraction and subsequent analysis phases. The articles retrieved from the databases were systematically arranged within an Excel spreadsheet, and meticulous steps were taken to eliminate any duplicate entries. In a collaborative effort, three authors independently reviewed the abstracts of these articles, leading to the identification of a subset of relevant papers. Following this, the full texts of these selected papers underwent a thorough individual assessment by the same authors, who then finalized the selection of pertinent articles.

After a thorough review of the selected articles, the three authors collaborated and reached a consensus by aligning their findings and assessments through discussions and adhering to the predefined inclusion and exclusion criteria.

## 3. Results

**Figure 4:**
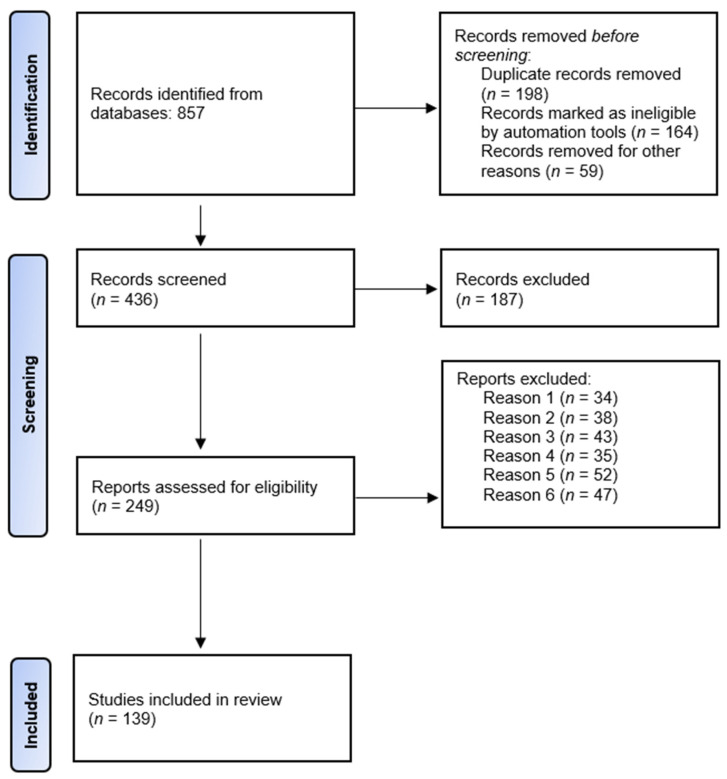
Flowchart of the selection process.

The initial search process yielded 857 articles based on their titles across the four chosen databases. After removing duplicates, 436 unique studies remained. Upon reviewing the abstracts, 187 articles were excluded either due to a lack of adequate data or differing study approaches. This left 249 full-length papers for comprehensive analysis. Ultimately, 139 of these full-text articles met the criteria for inclusion in this systematic review. Figure 4 illustrates a PRISMA flow chart that visually depicts the process of selecting studies for the review. This chart outlines the progression from the initial identification of articles to the final inclusion of studies in the systematic review. It provides a clear overview of this study’s selection process.

### 3.1. Dental Pulp-Dentin Regeneration

Dental pulp, a connective tissue located within teeth, is primarily composed of collagen fibers, proteoglycans, and various cell types, including fibroblasts, odontoblasts, and immune cells. Its viscoelastic attributes have been extensively documented [1,8,35,36]. Recent approaches, such as regenerative endodontic procedures, seek to stimulate the differentiation of resident or transplanted stem/progenitor cells to regenerate the dentin-pulp complex. Hydrogel-based scaffolds, characterized by high water content and a three-dimensional polymeric network structure, have emerged as promising platforms for this purpose. These hydrogels are biocompatible and hydrophilic in nature and offer tunable degradation patterns, mechanical properties, and the capacity to incorporate bioactive molecules. Their flexibility and elasticity resemble the extracellular matrix, particularly that of the dental pulp [37,38,39,40].

Dentin, the mineralized structure that surrounds dental pulp, consists of hydroxyapatite and an organic matrix primarily made up of collagenous and non-collagenous proteins [1,6]. Demineralized dentin matrix (DDM), obtained from human teeth, serves as an exceptional scaffold material with bioactive properties that can improve bone and dental tissue engineering. Promising outcomes have been reported for dentin, pulp, and periodontal regeneration using DDM [41].

Study models for pulp regeneration, as described by Ohlsson et al., can be categorized into in vitro, in vivo ectopic, in vivo semiorthotopic, and in vivo orthotopic approaches. These models encompass diverse strategies involving scaffold and cell transplantation in various animal contexts [42].

Addressing deep carious lesions, which often lead to irreversible pulpitis, traditional endodontic treatment involves the removal of the entire dental pulp tissue, potentially diminishing the tooth’s lifespan. Tissue engineering approaches offer an alternative by preserving the tooth function. Pulp revascularization is a regenerative endodontic treatment approach primarily aimed at promoting the reestablishment of blood supply and tissue regeneration in immature permanent teeth that have been affected by infected necrotic pulp and apical periodontitis. This technique involves disinfection, bleeding induction to create a regenerative niche, and subsequent hard tissue deposition. However, while it promotes root development, it primarily results in bone, cementum, and periodontal ligament-like fibrous tissue rather than true pulp regeneration [43,44,45,46].

Bioprinting advancements are significant in this context. Campos et al. successfully bioprinted bioinks containing cells and collagen, designed to have appropriate rheological, structural, and biological characteristics, to facilitate vasculogenesis within root canals. This approach showcases the potential for root canal vasculogenesis comparable to that of non-bioprintable controls [47].

Despite limitations associated with the resolution of extrusion-based 3D printing systems, encouraging outcomes have been observed in the application of these systems in regenerative dentistry. Although the clinical use of fiber-based cell-laden biomaterials for pulp-like tissue constructs remains a distant goal, the ability to pattern tissue structures in vitro presents an opportunity to develop intricate model systems that can provide deeper insights into physiological and pathological processes [48,49,50,51].

**Figure 5:**
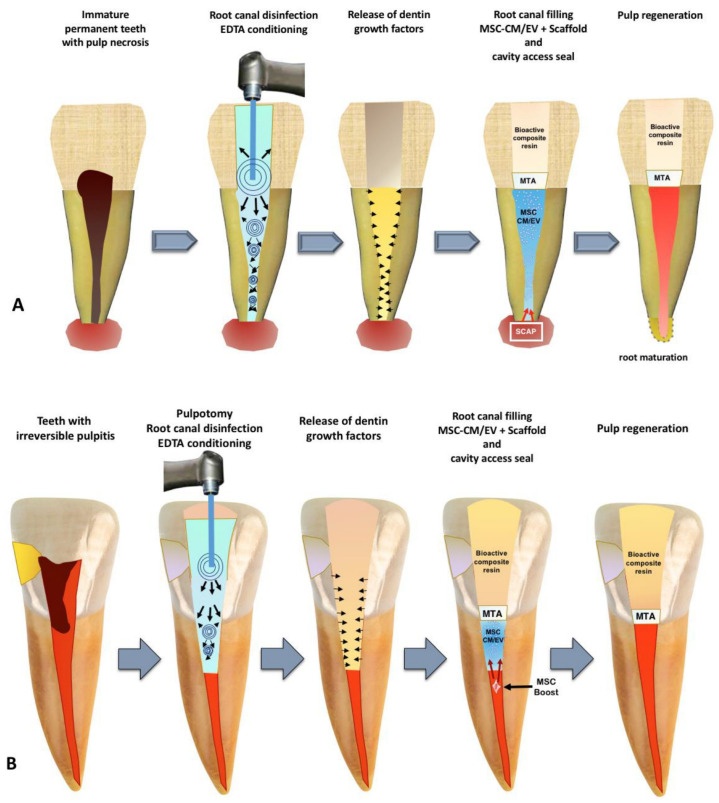
A summary of the proposal for a cell-free approach to dental pulp regeneration using MSC-derived CM/EV: (**A**) The treatment sequence for immature permanent teeth with pulp necrosis is outlined; (**B**) The regenerative procedure for cases with partially damaged pulp is summarized. Key components involved include: MTA (mineral trioxide aggregate), SCAP (stem cells from apical papilla), EDTA (ethylenediaminetetraacetic acid) [53].

In 2020, Brizuela and colleagues presented groundbreaking clinical evidence endorsing the use of allogenic umbilical cord mesenchymal stem cells enclosed within a plasma-derived biomaterial in endodontics. This approach, grounded in biological principles that support dentin-pulp regeneration, offers a promising alternative for treating periapical pathology. The incorporation of biological materials and stem cells into endodontic strategies offers the potential for improved treatment outcomes [52]. For cases of partial pulp damage, the stimulation of dental pulp stem cells (DPSCs) through mesenchymal stem cell (MSC)-derived secretome has shown promise in contributing to tissue formation and the restoration of dental pulp vasculature and nerves, Figure 5 [53,54,55]. 

Platelet-rich fibrin (PRF), employed either alone or in conjunction with bone grafts, has the capacity to promote bone growth and vascularization. Acting as a matrix for tissue ingrowth, PRF facilitates migration, cell attachment, and osteoblast proliferation, ultimately fostering bone formation. Its applications in regenerative endodontics include repairing iatrogenic perforations of the pulpal floor and revascularizing immature permanent teeth with necrotic pulps. This technique has yielded positive outcomes, including dentinal wall thickening, root lengthening, periapical lesion regression, and apical closure. However, further studies are necessary to clarify the precise mechanisms of the fundamental role of PRF in dental pulp regeneration, both in vitro and in vivo [56,57,58,59].

In 2021, Soares et al. emphasized the existing limitations of current clinical treatments, which often focus on managing the consequences of pulp exposure rather than actively restoring healthy dental pulp function. Repairing hard tissue-based dental structures presents challenges due to the inadequate self-healing capacity of enamel, partial restrictions on dentin and cementum regeneration, and complexities in achieving successful functional pulp regeneration [60]. In addition, the challenges inherent in repairing hard dental tissues are underscored, with the self-healing capabilities of enamel proving inadequate and the regeneration of dentin and cementum partially limited [5].

### 3.2. Bone Regeneration

Advancements in bone regeneration involve the integration of biological agents, such as osteogenic growth factors and mesenchymal stem cells (MSCs), into clinical applications. This integration has led to tangible and significant results [61,62,63]. Numerous strategies have emerged to promote osteogenesis, including bone grafts, scaffolds, stem cells, and growth factors [4,15,64,65,66,67].

Scaffolds designed for optimal bone regeneration should exhibit osteoconductive properties by supporting bone-forming cells, nourishing them, facilitating vascularization, and releasing signaling molecules that prompt mesenchymal stem cell differentiation and mineralized bone matrix deposition. Varied scaffold compositions, innovative technologies, signaling molecule incorporation, and high-quality biomaterials contribute to the successful osteoblastic differentiation of stem cells both in vitro and in vivo [68,69,70,71,72].

Significant genetic differences between alveolar bone and long bone have been elucidated by Son et al., underscoring the necessity for tissue-specific bone treatments. Alveolar bone requires a tailored treatment because of its unique characteristics [73].

Various scaffolds composed of distinct materials, including metals, ceramics, and polymers, have been employed to ensure specific mechanical attributes of substrates [3,74,75]. An intriguing clinical approach in the realm of bone tissue engineering involves the integration of these scaffolds; however, it is important to consider their potential to induce adverse effects on cell-cell interactions. In addition, the application of scaffold materials for bone tissue reconstruction necessitates the preliminary manipulation of cells using proteolytic enzymes such as trypsin or dispase. Regrettably, this enzymatic treatment can lead to cellular damage and a loss of cellular functionality due to the degradation of extracellular matrix (ECM) molecules and cell surface proteins. Consequently, the field demands innovative strategies for promoting bone regeneration that circumvent these challenges. In recent times, a pioneering tissue engineering methodology termed ‘cell sheet’ has emerged as an effective solution for the regeneration of various tissues, such as bone, corneal, cardiac, tracheal, and periodontal ligament-like tissues. The fundamental concept of the cell sheet involves a coherent layer of cells, replete with intact ECM and essential cell surface proteins, including receptors for growth factors, ion channels, and intercellular junction proteins. Notably, mesenchymal stem cell (MSC) sheets can be conveniently fabricated by layering retrieved cell sheets without the need for complex scaffolding or intricate manipulation [76,77,78].

Hasani-Sadrabadi et al. devised an injectable adhesive hydrogel based on alginate. The hydrogel contained clusters of gingival mesenchymal stem cells and osteoconductive hydroxyapatite microparticles. In a rat model designed to simulate peri-implantitis, this hydrogel formulation promoted the regeneration of bone around dental implants. Notably, the hydrogel’s degradation rate and mechanical properties could be finely controlled, indicating its potential as a valuable tool in tissue engineering [79]. The prominence of hydrogel-based scaffolds has garnered considerable attention, chiefly because of their distinctive attributes, such as safety, compatibility with biological systems, cost-effectiveness, ease of production and customization, versatile application potential, simplicity in various synthesis methods, and effective cellular adhesion facilitated by their natural components. Hydrogels have demonstrated their utility as delivery vehicles for stem cells, notably human dental pulp stem cells (hDPSCs), for the restoration of damaged dentin and pulp tissues [80,81,82,83,84]. Advances in craniofacial tissue and organ regeneration have shown promise through the use of bioinks containing different biomaterials and integrated stem cells within 3D bioprinting platforms. Although strides have been made in fabricating craniofacial bone and cartilage structures, the pursuit of achieving optimal outcomes remains ongoing [85,86]. Calcium phosphate has served as a pivotal element in various forms for bone regeneration, encompassing coatings, cements, and scaffold structures, owing to its distinctive bioactive characteristics and effectiveness in promoting bone regeneration. An innovative approach involved the combination of tetracalcium phosphate and dicalcium phosphate anhydrate with alginate hydrogel microbeads that encapsulated human umbilical cord mesenchymal stem cells. This strategy aimed to address the limitations of mechanical strength within the hydrogel for load-bearing applications, along with the challenges of cell seeding in the scaffold’s interior and injectability during minimally invasive surgeries. The resultant injectable alginate hydrogel scaffold exhibited augmented mechanical properties compared with conventional hydrogels [87,88,89,90]. Prior research in the realm of bone regeneration has underscored the favorable effects of zinc and magnesium ions present in bioactive glass compositions. However, an explicit exploration of the influence of these bioactive glasses on polymer matrix composites remains a lacuna. The proposed methodology focuses on enhancing antibacterial efficacy, biological activity, and mechanical attributes of composite bone scaffolds through the integration of zinc- and magnesium-containing bioactive glasses within alginate networks [91,92,93,94,95].

Collagen is a vital macromolecule found within the extracellular matrix (ECM) of bones, teeth, and temporomandibular joints. It plays a pivotal role in preserving the structural integrity and functionality of these tissues. In the context of regenerative dentistry, synthetic biomaterials based on collagen hold promise as effective scaffolds. These biomaterials replicate the composition of the host tissues’ ECM, making them conducive for applications in tissue regeneration. Notably, collagen-based biomaterials possess attributes such as biocompatibility, biodegradability, ready availability, and non-toxicity to cells. These characteristics foster favorable cellular responses and tissue repair within the craniofacial area. Furthermore, collagen can be engineered to incorporate additional biomolecules, enabling the induction of mineralization in tissues like bone and teeth. The augmentation of collagen-based biomaterials with such molecules or other polymers can lead to improvements in mechanical properties, a critical aspect in load-bearing regions such as the mandible [4,96,97,98,99].

Effective bone tissue engineering (BTE) using stem cells requires a trifecta of components: an ample supply of mesenchymal progenitors with osteogenic potential, appropriate bioactive factors to guide osteogenic differentiation, and scaffold biomaterials that facilitate cell interactions. The linchpin of BTE lies in the selection of scaffold biomaterials that are conducive to cellular adherence and proliferation, addressing extensive bone defects [10,100,101,102,103]. In the pursuit of reconstructing alveolar bone tissue, tissue engineering strategies and stem cell-based regenerative therapies are promising alternatives. The success of these approaches hinges on assembling the optimal combination of cells, scaffolds, signaling molecules, and nanomaterials. Nanomaterials, characterized by their exceptional physicochemical properties and biomimetic attributes, have demonstrated the potential for promoting cell growth and eliciting tissue regeneration. Within the realm of oral tissue engineering, the integration of nanomaterials holds significant promise as a future treatment modality [104,105,106,107,108].

Given the highly vascular nature of bone tissue, recent research efforts have been directed towards innovative strategies centered around the development of pre-vascularized scaffolds or pre-vascularized cellular aggregates. These approaches have become a recurring theme in scientific investigations. In the realm of bone tissue engineering, two primary paradigms are distinguished: biomimetic scaffold-based approaches and scaffold-free methods. The emphasis of these approaches lies in the choice of materials and fabrication techniques employed. A central aspect of exploration involves the biomimetic vascularized strategies, which encompass the creation of pre-vascularized scaffolds and pre-vascularized cellular aggregates [109,110,111,112,113].

Leveraging accessible technology, specialized 3D bioprinters with the capability to produce sophisticated bioinks have been harnessed for the fabrication of biosensors. These 3D-printed biosensors serve to regulate conductivity and electrical transmission within physiological environments. The integration of stem cell-containing scaffolds into the printed constructs yields substantial effects on cellular behavior and differentiation [114,115,116].

### 3.3. Periodontium Regeneration

Periodontitis, a detrimental pathological state, has a significant influence on both the soft and hard tissues that surround the tooth. The foremost categories of biomaterials employed in the context of periodontal regeneration encompass barrier membranes, grafting materials, biological agents, and, more recently, three-dimensional (3D) scaffolds [117,118,119].

During the previous twenty years, various regenerative strategies have been developed for the treatment of periodontal conditions. These include guided tissue regeneration (GTR), the use of derivatives from enamel matrix, bone grafting, the delivery of growth factors, as well as the incorporation of cells and growth factors into scaffolds based on matrices. These methods target the restoration of tooth-supporting tissues that are lost due to periodontal disease, encompassing the periodontal ligament, alveolar bone, and cementum [2,120,121,122]. Scaffolds for periodontal regeneration, whether monophasic or multiphasic, can be 3D printed using various materials like natural polymers, synthetic polymers, or bioceramics. This process relies on computer-assisted design and manufacturing, often guided by CT scans for personalized scaffold creation. To boost tissue regeneration and increase bioactivity, stem cells and/or growth factors can be incorporated into these scaffolds, aiming to replicate the intricate architecture of periodontal tissue [123,124,125,126]. Despite the strides made in scaffold fabrication and their evident efficacy in guiding and supporting tissue regeneration, the availability of appropriate cell sources remains a critical factor for driving new tissue formation. Additionally, the extracellular matrix (ECM) provides a multitude of biological and mechanochemical cues necessary for stimulating cell growth and differentiation [127,128,129,130]. Notably, the cultivation of periodontal ligament (PDL) cell spheroids within 3D-printed polylactic acid scaffolds has demonstrated enhanced migration ability compared to 2D monolayer cells. This implies that inducing spheroid formation of periodontal ligament (PDL) cells through biomaterials could be a novel approach for delivering cells in research and clinical contexts, with the aim of promoting periodontal regeneration [131,132]. Innovative efforts have been directed at designing fibrous scaffolds that mimic the structure of the periodontal ligament (PDL) matrix, along with the integration of human PDL fibroblasts (PDLFs). These scaffolds, cultured with PDLFs, mirror the morphological traits observed in native PDLFs and evoke proliferative, osteoblastic, and osteoclastogenic potentials based on fiber topographical cues [133,134].

The fiber-reinforced hydrogel approach involves the combination of porous poly(ε-caprolactone) (PCL) fibrous meshes with a three-dimensional (3D) structure within bioactive amorphous magnesium phosphate-laden gelatin methacryloyl hydrogels. This composite system takes advantage of the presence of amorphous magnesium phosphate and PCL mesh to effectively control mechanical properties and enhance osteogenic (bone-forming) potential. This innovation shows great promise in the field of guided bone regeneration (GBR). The research findings indicate that incorporating PCL meshes, created using melt electro writing, serves to hinder the degradation of the hydrogel, preventing the infiltration of soft tissue. Furthermore, the PCL mesh acts as a mechanical barrier, facilitating the involvement of slower-moving progenitor cells in bone regeneration and their subsequent transformation into bone-forming cells [135,136,137,138,139]. In contrast to cell-based therapeutic strategies, biomaterial-based approaches offer a comparably straightforward and reliably supportive means for substantial endogenous tissue regeneration. As such, endogenous regenerative technology has emerged as a budget-friendly option, efficient, and secure method for clinical patient treatment. In the context of periodontal regeneration, tissue engineering strategies can be broadly categorized as scaffold-free or scaffold-based [140,141]. Xu et al. demonstrated the creation of a 3D multilayered scaffold by assembling and securing electrospun polycaprolactone/gelatin (PCL/Gel) fibrous membranes. This biomaterial exhibited favorable hydrophilic and mechanical traits. Significant results included not just the regeneration of new bone but also the appearance of angular, concentrated fiber regeneration on the root surface of the defect. This closely resembled the structure of normal periodontal tissue [142].

Bioactive factors play a pivotal role in periodontal regeneration. These molecules function as essential regulators in various aspects of the regenerative process. They influence the differentiation of precursor cells into specific periodontal tissues, stimulate resident stem cells to migrate to damaged sites, and attract immune cells to modulate the inflammatory response, thereby fostering the regeneration of periodontal tissues [17,136,140,143]

Five distinct types of stem cells have been recognized as potential candidates for use in periodontal regeneration: periodontal ligament-derived stem cells (PDLSCs), bone marrow-derived stem cells (BMSCs), adipose tissue-derived stem cells (ADSCs), dental pulp-derived stem cells (DPSCs), and gingival-derived stem cells (GMSCs). Among these, PDLSCs and BMSCs have demonstrated superior efficacy and are well-documented candidates for periodontal tissue regeneration [5,7,8]. PDLSCs and BMSCs are emerging as particularly promising candidates without statistically significant differences in their regenerative potential [144].

Nagayasu-Tanaka et al. conducted research to investigate the combined effects of fibroblast growth factor-2 (FGF-2), a drug used for periodontal regeneration and carbonated apatite (CO3Ap), a bioresorbable and osteoconductive scaffold. Their study specifically centered on periodontal regeneration within one-wall periodontal defects. The results of their study showed that the combination of FGF-2 and CO3Ap not only promoted increased formation of new bone and scaffold replacement but also preserved the integrity of the adjacent bone near the defect site [145].

Because of its inherent biocompatibility and flexibility, collagen (Col) is extensively employed in tissue engineering and medical applications. Col sponges possess the ability to absorb growth factors, expediting the periodontal tissue regeneration process. The malleability, low immunogenicity, and hemostatic properties of Col enable its integration into the oral tissue, enabling cells with regenerative potential to populate defected regions [146,147,148].

However, achieving comprehensive regeneration of periodontal supporting tissues remains a formidable challenge within current technological constraints. In the realms of in vitro cell-biomaterial design and transplantation, further exploration is required to refine biomaterial devices that can effectively harness the innate regenerative capacities of the periodontium [99,143]. The design of cell-biomaterial interactions in vitro and the subsequent transplantation of engineered biomaterial devices represent areas of significant promise for advancing periodontal regeneration. This approach aims to capitalize on the inherent regenerative potential of the periodontium, and as such, calls for continued and thorough investigation [142].

### 3.4. Gingival Regeneration

Very little attention is paid to the soft tissue repair of the gingiva. For the last 5 years, only nine articles mention “gingival regeneration”, three of which were not appropriate. Many discussions often categorize it within the broader context of periodontal regeneration [1,149,150].

Therapeutic strategies for addressing gingival recession have traditionally focused on periodontal plastic surgery, specifically for tasks such as root coverage and reconstruction of the enamel-cement junction (CEJ). While nanomaterial-based transplants offer a potential solution for covering and protecting alveolar bone defects, the direct exposure of the gingival wound or recessive gingiva to the intricate oral microenvironment poses challenges. The application of nanomaterials could potentially alter the gingiva’s color, shape, and texture, significantly impacting smile aesthetics, especially in the context of anterior teeth [151].

Hydrogel materials are gaining recognition as highly promising scaffold biomaterials for promoting gingival regeneration. In vitro experiments play a crucial role as testing grounds for evaluating potential biomaterials that could eventually be applied in clinical practice. Researchers have been investigating the incorporation of natural polymers, like collagen, chitosan, and hyaluronic acids, to enhance the properties of these biomaterials. However, the use of synthetic polymers has faced limitations due to their physical and biological characteristics. To enhance cell adhesion and migration, peptides, such as growth factors and arginine-glycine-aspartic acid (RGD), can be employed. These strategies aim to improve the effectiveness of hydrogel-based materials in promoting gingival tissue regeneration [152]. Chitosan hydrogels play a critical role in vascular remodeling and regeneration through controlled drug and growth factor release, thus contributing to tissue vascular regeneration [153].

Table 1 in this document presents a synthesis of the scientific literature, highlighting the most significant directions in dental tissue bioprinting. This table likely provides a summary of key findings and research directions in the field of bioprinting for dental tissue regeneration, making it a valuable reference for readers looking for an overview of the current condition of research in this field.

## 4. Discussion

Significant strides have been achieved in the realms of 3D printing and bioprinting, with these technologies finding applications across diverse fields, including tissue engineering, regenerative medicine, personalized medicine, prostheses, implants, drug manufacturing, and medical education [1,7].

Dental pulp stem cells (DPSCs) exhibit a promising potential as an alternative therapeutic approach for addressing neurodegenerative diseases. Various factors influence the osteogenic and neurogenic differentiation capabilities of DPSCs, with careful consideration required to balance positive and negative influences. Precise control over culturing methods, co-factor supplementation, and synthetic or natural environments enables the expansion of DPSCs while retaining their stemness properties. Immunophenotyping and cell sorting technologies contribute to enhancing the quality of DPSC populations derived from pulp tissue. However, the limited experimental evidence concerning cell migration hinders comprehensive comprehension and assessment of cell therapy efficacy, underscoring the need for further investigation [154].

Dental follicle progenitor cells (DFPCs) serve pivotal roles during tooth development. They assist in tooth eruption by providing traction and facilitating the creation of a pathway for eruption, influencing alveolar bone formation and resorption. Furthermore, DFPCs are instrumental in the development and maintenance of the periodontal attachment apparatus, which is crucial for sensing mechanical stress and ensuring the tooth’s physiological function [155]. Dental follicle cells (DFCs), a subset of mesenchymal progenitor cells located around the tooth germ, also play a significant role in tooth development. They contribute to the formation of essential structures, like cementum, periodontal ligament, and alveolar bone. Signaling pathways and transcription factors within DFCs can coordinate processes, such as tooth eruption and root morphogenesis. DFCs exhibit versatile characteristics, with the ability to differentiate into various cell types, including osteoblasts/cementoblasts, adipocytes, and neuron-like cells. This multipotency makes DFCs well-suited for clinical applications, such as bone tissue engineering, tooth root regeneration, and periodontium regeneration. Beyond their applications in oral and maxillofacial regeneration, DFCs show potential for use in other areas, such as addressing spinal cord defects and repairing damaged cardiomyocytes. Their versatility and regenerative potential make them a promising focus of research with wide-ranging implications in regenerative medicine [156].

The field of 3D bioprinting relies on cell-laden bioinks and encompasses diverse techniques and strategies. However, challenges are evident when compared to non-biological 3D printing approaches, particularly in constructing bioscaffolds that closely mimic native tissues [4]. Periodontal regeneration through the implementation of controlled drug delivery and the utilization of biomaterials encompasses a range of inorganic, polymeric, or composite biomaterials. The application of inorganic biomaterials is particularly beneficial for the restoration of bone and cementum, as their composition and mechanical properties closely resemble those of natural tissue. For periodontal ligament (PDL) regeneration, polymeric biomaterials are more suitable. The process of creating artificial scaffolds that imitate the properties of natural bone and cementum for the purpose of regenerating these tissues requires the combination of inorganic and polymeric biomaterials [117,141]. The integration of dentin-derived matrix (DDM) into other biomaterials has proven to be highly beneficial. By harnessing DDM’s natural growth factors and nano-minerals, this approach has been effective in stimulating both bone and dental regeneration processes [41].

Nonetheless, challenges persist. Ensuring that the activation of signaling pathways remains physiological, developing controlled drug release systems, and assessing the safety of gene modulation are crucial aspects that warrant exploration for effective clinical translation of pulp regeneration [157]. Micro- and nanofabrication techniques continue to be subjects of discussion in various domains of dental research, including endodontic and periodontal regeneration, biomaterials research, dental implantology, oral pathology, and biofilms. There is a growing exploration of how these approaches could soon find widespread use in clinics for the diagnosis, prevention, and treatment of relevant dental pathologies [158].

Given the intricate interrelationships among dental alveolar tissues, the utilization of multimaterial and multicellular bioprinting often becomes imperative [1].

The advancement of regenerating complete teeth necessitates comprehensive scientific exploration across various levels. This involves the search for appropriate cell sources that carry tooth-inductive signals and delving deeper into the potential of induced pluripotent stem (iPS) cells for this role. Additionally, comprehending the master genes within gene regulatory networks responsible for tooth induction and formation is crucial. This understanding is essential for efficiently guiding adult cells to create bioengineered dental tissues and for controlling critical aspects such as tooth crown development, size, and identity [159].

Addressing the limited regenerative capacity of the periodontium necessitates the creation of novel biomaterials and therapeutic strategies. It is worth emphasizing that the periodontium’s regenerative potential is closely tied to its distinctive tissue architecture, function, and ability to rebuild various tissues and tissue interfaces. This indicates that progress in tissue engineering techniques offers the potential to support organized reconstruction of both the soft and hard tissues within the periodontium [139].

Additionally, stem cells from the apical papilla (SCAP) exhibit the expression of various neurogenic markers, like nestin and neurofilament M when exposed to a neurogenic medium. While SCAP share similarities with dental pulp stem cells (DPSCs), they represent a unique source of highly potent dental stem/progenitor cells. The significance of SCAP in root development and apexogenesis is a topic of exploration and discussion [31].

Regenerative medicine has ushered in new possibilities for dentoalveolar treatments, especially in cases where traditional approaches fall short. Nonetheless, the translation of tissue regeneration strategies into clinical practice remains somewhat limited. Challenges include the complexity of replicating intricate tissue architectures and the variability inherent in the regeneration process, often resulting in biologically insignificant outcomes. Bioprinting, however, holds promise in tackling some of these challenges. The selection of appropriate materials that facilitate cell encapsulation and are compatible with bioprinting processes stands as a major hurdle in bioprinting dentoalveolar tissues. While the field of bioprinting dentoalveolar tissue is still in its nascent stages, there exists a wide scope for further exploration [6].

Kim et al. highlight the promising potential of utilizing biopolymers in the field of bioprinting for dental tissue engineering, emphasizing the integration of polymeric biomaterials and bioprinting techniques that have yielded significant advancements in the regeneration of dental tissues, like bone, periodontal ligament, and dentin; furthermore, the article suggests that to further advance this field, future research should focus on the convergence of functional 3D bioprinting with advanced imaging technologies and the adoption of autologous tissue implantation, ultimately offering innovative strategies that hold substantial promise for enhancing dental tissue regeneration and potentially revolutionizing clinical practices [160].

While there has been progress, challenges related to the bioavailability of bioinks, mechanical properties, dimensional accuracy, and translating models to human subjects remain. Continued improvement in digitally assisted techniques, biomaterials, and the integration of patient-specific data can facilitate translation to clinical applications [124]. It is essential to thoroughly investigate the potential side effects of stem/progenitor transplantation before implementing them as clinical therapies in restorative dentistry [161].

Although 3D printing costs have reduced over time, there are still financial considerations related to materials, equipment maintenance, and the need for skilled professionals. Strict adherence to safety protocols is also vital. However, as the technology continues to develop, it is expected to play an increasing role in dentistry [162].

Of course, many limitations related to the bioavailability of bioinks, the mechanical properties of the printed structure and its dimensional accuracy are still to be overcome. Moreover, translating the obtained models into human subjects remains an important challenge. Improving digitally assisted techniques and biomaterials, together with the combination of CBCT investigations, can facilitate this translation, with the production of patient- and site-specific scaffolds [124].

## 5. Conclusions

Indeed, it is still early to definitively discuss the long-term benefits of bioprinting, especially in the challenging context of the maxillofacial area. This region encompasses a wide range of diverse tissues, each with a complex innervation and blood supply. It is also home to important sensory organs and vital structures like the brain. The intricate and multifaceted nature of the blood supply makes it difficult to experiment with arbitrary attempts.

While there is foundational theoretical knowledge available in the scientific literature, clinical trials are relatively scarce, and most of them lack comprehensive information on long-term outcomes. Bioprinting’s potential benefits are often emphasized, but there is a dearth of preclinical and clinical studies that delve into the difficulties, side effects, and limitations of applying bioprinting techniques in dentistry.

The field of bioprinting in dentistry is still in its early stages, and many unknowns remain to be explored. Research and development efforts are ongoing to address these challenges and gain a better understanding of the technology’s true potential and limitations in the context of dental and maxillofacial applications.

## 6. Limitations

This systematic review has several limitations that warrant consideration. Firstly, there is the possibility of publication bias due to the study’s exclusive focus on English-language articles published between 2019 and 2023, potentially leading to the omission of relevant research in other languages. Additionally, the temporal restriction of the search for articles up to September 2023 may result in the omission of recent developments. Despite a comprehensive search strategy, it is possible that some relevant articles remain undiscovered. The synthesis and interpretation of data may be subject to bias due to the diversity of objectives and methodologies of the included articles. Therefore, the risk of bias assessment was not performed. Furthermore, the generalizability of the findings may be limited, given the diverse landscape of bioprinting technologies and dental applications.

## Figures and Tables

**Table 1:** Significant directions in dental tissue bioprinting.

Key Benefit/Topic	Area of Application/Significance	References
Dental pulp-dentin regeneration	3D bioprinted scaffolds have potential to stimulate the differentiation of resident or transplanted stem/progenitor cells to regenerate the dentin-pulp complex	Abbass et al., 2020; Xie et al., 2021; Ayala-ham et al., 2021; Sugiaman et al., 2023 [37,38,39,40]
	Realm of dentin, pulp, and periodontal regeneration using Demineralized dentin matrix	Gao et al., 2019 [41]
	Tissue engineering approaches offer pulp revascularization as an alternative by preserving tooth function.	Thalakiriyawa& Dissanayaka, 2023; Wei et al., 2022; Bertassoni, 2020; Widbiller & Galler, 2023 [43,44,45,46]
	Bbioprinted cell-loaded collagen-based bioinks showcases potential for root canal vasculogenesis	Campos et al., 2020 [47]
	3D bioprinted materials conduce to dentin-pulp regeneration	Brizuela et al., 2020; Costa et al., 2022; Dolega-Dolegowski et al., 2023; Iandolo, 2023; Arshad et al., 2021; Wu et al., 2023; Tang et al., 2022; Heboyan et al., 2022 [52,53,54,55,56,57,58,59]
Bone regeneration	Hydrogel based on alginate facilitated the regeneration of bone around dental implants	Hasani-Sadrabadi et al., 2020 [79]
	Bioinks and hydrogels have the potential for the restoration of damaged dentin and pulp tissues	Samiei et al., 2021; Gao et al., 2021; Lin et al., 2019; Vurat et al., 2023; Van Hede et al., 2022 [80,81,82,83,84]
	3D bioprinting platforms have the potential for fabricating craniofacial bone and cartilage structures	Dwivedi & Mehrotra, 2020; Atia et al., 2023 [85,86]
	Biomaterials for of scaffold that are conducive to cellular adherence and proliferation, have potential to lead addressing extensive bone defects	Zhang, et al., 2020; Zhang, et al., 2020; Iaquinta et al., 2019; Loukelis et al., 2023; Gan et al., 2020 [10,100,101,102,103]
Periodontium regeneration	Three-dimensional printing of scaffolds has emerged as a compelling alternative to traditional periodontal regeneration methods	Raveau & Jordana, 2020; Sufaru et al., 2022; d’Avanzo et al., 2021; Woo et al., 2021; Bousnaki et al., 2022; Yang et al., 2023; Liang et al., 2023; Miao et al., 2023 [123,124,125,126,127,128,129,130]
	Biomaterial-based approaches offer a comparably straightforward and reliably supportive means for substantial endogenous tissue regeneration	Xu et al., 2019; Matichescu et al., 2020 [140,141]
	Specific biomaterials have potential for new bone regeneration, and also for the emergence of angular, concentrated fiber regeneration on the root surface of the defect	Xu et al., 2020 [142]
	Bioactive factors influence the differentiation of precursor cells into specific periodontal tissues, stimulate resident stem cells to migrate to damaged sites, and attract immune cells to modulate the inflammatory response	Xu et al., 2019; Mancini et al., 2021; Liu et al., 2020; Zhai et al., 2019 [17,140,143,144]
Gingival regeneration	The application of nanomaterials could potentially alter the gingiva’s color, shape, and texture, significantly impacting smile aesthetics, especially in the context of anterior teeth	Zong et al., 2023 [151]
	Hydrogel materials are emerging as promising scaffold biomaterials for gingival regeneration	Hutomo et al., 2023 [152]

## Data Availability

Data are available from the corresponding author on request.
